# Impact of Age and Variant Time Period on Clinical Presentation and Outcomes of Hospitalized Coronavirus Disease 2019 Patients

**DOI:** 10.1016/j.mayocpiqo.2023.07.004

**Published:** 2023-09-15

**Authors:** Pratyaksh K. Srivastava, Alexandra M. Klomhaus, David M. Tehrani, Gregg C. Fonarow, Boback Ziaeian, Pooja S. Desai, Asim Rafique, James de Lemos, Rushi V. Parikh, Eric H. Yang

**Affiliations:** a Ahmanson-UCLA Cardiomyopathy Center, Ronald Reagan UCLA Medical Center, Los Angeles, CA; b Department of Medicine, Statistics Core, UCLA, Los Angeles, CA; c Division of Cardiology, Ronald Reagan UCLA Medical Center, Los Angeles, CA; d Division of Cardiology, West Los Angeles Medical Center, Los Angeles, CA; e Department of Cardiology, Smidt Heart Institute, Cedars-Sinai Medical Center, Los Angeles, CA; f Division of Cardiology, Santa Monica UCLA Medical Center, Los Angeles, CA; g Division of Cardiology, UT Southwestern Medical Center, Dallas, TX

## Abstract

**Objective:**

To evaluate the impact of age and COVID-19 variant time period on morbidity and mortality among those hospitalized with COVID-19.

**Patients and Methods:**

Patients from the American Heart Association’s Get With The Guidelines COVID-19 cardiovascular disease registry (January 20, 2020-February 14, 2022) were divided into groups based on whether they presented during periods of wild type/alpha, delta, or omicron predominance. They were further subdivided by age (young: 18-40 years; older: more than 40 years), and characteristics and outcomes were compared.

**Results:**

The cohort consisted of 45,421 hospitalized COVID-19 patients (wild type/alpha period: 41,426, delta period: 3349, and omicron period: 646). Among young patients (18-40 years), presentation during delta was associated with increased odds of severe COVID-19 (OR, 1.6; 95% CI, 1.3-2.1), major adverse cardiovascular events (MACE) (OR, 1.8; 95% CI, 1.3-2.5), and in-hospital mortality (OR, 2.2; 95% CI, 1.5-3.3) when compared with presentation during wild type/alpha. Among older patients (more than 40 years), presentation during delta was associated with increased odds of severe COVID-19 (OR, 1.2; 95% CI, 1.1-1.3), MACE (OR, 1.5; 95% CI, 1.4-1.7), and in-hospital mortality (OR, 1.4; 95% CI, 1.3-1.6) when compared with wild type/alpha. Among older patients (more than 40 years), presentation during omicron associated with decreased odds of severe COVID-19 (OR, 0.7; 95% CI, 0.5-0.9) and in-hospital mortality (OR, 0.6; 95% CI, 0.5-0.9) when compared with wild type/alpha.

**Conclusion:**

Among hospitalized adults with COVID-19, presentation during a time of delta predominance was associated with increased odds of severe COVID-19, MACE, and in-hospital mortality compared with presentation during wild type/alpha. Among older patients (aged more than 40 years), presentation during omicron was associated with decreased odds of severe COVID-19 and in-hospital mortality compared with wild type/alpha.

Severe Acute Respiratory Syndrome Coronavirus 2 (SARS-CoV-2), the virus responsible for the Coronavirus Disease-2019 (COVID-19) pandemic, was first discovered in Hubei Province, China, in late 2019.^1^ Since then, the virus has spread rapidly around the globe. The initial strains discovered included the wild type and alpha variants (B 1.1.7). The delta variant (B.1.617) was discovered in India in late 2020 and became the dominant strain in the United States around the beginning of July 2021. More recently, the omicron variant was discovered in South Africa in November 2021, and became the dominant variant in the United States at the end of 2021.^2^^,^^3^

Previous studies have found considerable differences between these 3 predominant variants. For example, the delta variant has been shown to be more transmissible and to cause more severe disease when compared with wild type, whereas the omicron variant is associated with decreased risk of hospitalization and less severe outcomes when compared with delta.^4^, ^5^, ^6^, ^7^ Throughout the pandemic, age has also emerged as a significant predictor of COVID-19 outcomes. For example, a large study evaluating the impact of age across 45 countries reported a log linear increase in the infection fatality ratio in those older than 30 years.^8^ In another study of 5279 people from New York city, age was the strongest risk factor for hospital admission and among the strongest predictors of critical illness.^9^

Although previous studies have compared one variant to another, few have compared all major variants to each other in a national population. Furthermore, few large studies have evaluated the differential impact of age across all 3 variants. Herein, we address this evidence gap and utilize a large national US database to evaluate the impact of age and variant time period on patient characteristics, treatment patterns, and clinical outcomes among patients hospitalized with COVID-19.

## Methods

The American Heart Association’s (AHA) Get With The Guidelines (GWTG) COVID-19 cardiovascular disease registry was created to serve as an in-patient data repository for hospitalized adult COVID-19 patients aged 18 years or older with the aim of supporting quality improvement and research. The registry was launched in April 2020 and includes 134 hospitals, health centers, and medical centers from 34 states across the United States. An institutional review board either waived review or approved patient enrollment at the participating centers. Full details of the registry have been previously described.^10^

Using the AHA’s GWTG COVID-19 registry, we identified patients hospitalized across the United States with a diagnosis of COVID-19. Patients were divided temporally into different COVID-19 variant time periods depending on their time of admission to the hospital. Patients admitted between January 20, 2020 and July 5, 2021 were grouped into the wild type/alpha period, between July 6, 2021 and December 27, 2021 into the delta period, and between December 28, 2021 and February 14, 2022 into the omicron time period. The alpha variant became the dominant strain in the United States around March 2021, though it did not cause a marked spike in new cases. The wild type and alpha waves were combined for the purposes of this analysis. Date cutoffs were chosen a priori on the basis of the date at which the particular variant became the predominant strain in the United States.^3^ Patient demographic characteristics, medical comorbidities, vitals on hospital presentation, admission symptoms, medications before admission, laboratory reports, therapies received during hospitalization, procedures performed during hospitalization, and outcomes during hospitalization were compared between age groups within the variant time periods and across time periods within age strata. Continuous and categorical variables were compared across the variants using Kruskal Wallis and χ^2^ tests, respectively.

Using adjusted logistic regression, we evaluated the association of COVID-19 variant time period with in-hospital patient outcomes. Models were run separately for those aged 18-40 years (young) and for those aged more than 40 years (older). Further models were run evaluating the impact of age category (older vs young) on in-hospital outcomes. Outcomes included severe COVID-19, major adverse cardiovascular events (MACE), thromboembolic disease (deep vein thrombosis or pulmonary embolism), and in-hospital mortality. Severe COVID-19 was defined as patients experiencing mechanical ventilation, cardiac arrest, or death while hospitalized. MACE was defined as a composite of myocardial infarction, new-onset heart failure, stroke, or death while hospitalized. Last, multivariate cubic spline models were created to continuously model the impact of age on predicted probability of severe COVID-19, MACE, and in-hospital mortality across all 3 COVID-19 variant time periods. Splines were constructed using 3 knots placed at evenly spaced percentiles. Logistic regression and spline models were adjusted for age (where appropriate), sex, body mass index, race or ethnic group, payment source, and medical comorbidities (atrial fibrillation or flutter, cancer, cerebrovascular disease, chronic kidney disease, congenital heart disease, coronary artery disease, diabetes mellitus, dyslipidemia, heart failure, hypertension, immune disorders, peripheral artery disease, pulmonary embolism, pulmonary disease, and smoking).

All statistical analyses were conducted using SAS on the AHA’s precision medicine platform.^11^ A 2-sided *P*<.05 was set as a threshold for statistical significance.

## Results

The overall cohort consisted of 45,421 patients hospitalized with a confirmed diagnosis of COVID-19 between January 20, 2020 and February 14, 2022. Of these, 41,426 were admitted during the wild type/alpha variant time period, 3349 during the delta period, and 646 during the omicron period. The median age (95% CI) of the cohort was 63 years (50-75), and 46.8% of the group was female. The general demographic characteristics and medical comorbidities stratified by age group (young: 18-40 years and older: more than 40 years) and variant time period are shown in Table 1. Of the overall hospitalized cohort, 21.4% of patients were non-Hispanic Black or African American, and 19.4% of the group was Hispanic. Hospitalized patients were more likely to be non-Hispanic Black or Hispanic, and less likely to be non-Hispanic White during the wild type/alpha period compared with the delta and omicron periods in both age groups (Table 1).

**Table 1:** Characteristics of the Cohort Stratified by Age and Coronavirus Disease 2019 Variant Time Period^a^

Characteristic	Overall	Wild type/alpha wave (January 20, 2020-July 5, 2021), n=41,426			Delta wave (July 6, 2021-December 27, 2021), n=3349			Omicron wave (December 28, 2021-February 14, 2022), n=646			Comparison *P* value^b^	Comparison *P* value^b^
	(January 20, 2020-February 14, 2022)	Young (18-40 y)	Older (>40 y)	*P* value	Young (18-40 y)	Older (>40 y)	*P* value	Young (18-40 y)	Older (>40 y)	*P* value	Young (18-40 y)	Older (>40 y)
	N=45,421	n=5585	n=35,841		n=690	n=2659		n=213	n=433			
Demographic characteristic^c^												
Age, y	63.0 (50.0-75.0)	32.0 (27.0-37.0)	67.0 (57.0-78.0)	N/A	32.0 (27.0-36.0)	63.0 (53.0-73.0)	N/A	30.0 (25.0-35.0)	63.0 (54.0-73.0)	N/A	.007	<.001
Female, n (%)	21,274 (46.8)	3015 (54.0)	16,298 (45.5)	<.001	384 (55.7)	1231 (46.3)	<.001	148 (69.5)	198 (45.7)	<.001	<.001	.91
Body mass index, kg/m^2^	29.8 (25.4-35.5)	32.1 (26.7-38.7)	29.4 (25.1-34.9)	<.001	32.6 (26.3-39.8)	30.4 (35.5-36.2)	<.001	29.9 (25.5-38.2)	28.8 (24.4-33.9)	.02	.025	<.001
Race, n (%)				<.001			<.001			<.001	<.001	<.001
Asian	1371 (3.0)	190 (3.4)	1137 (3.2)		12 (1.7)	13 (0.5)		8 (3.8)	11 (2.5)			
American Indian/Alaska Native	299 (0.7)	35 (0.6)	178 (0.5)		10 (1.4)	65 (2.4)		2 (0.9)	9 (2.1)			
Non-Hispanic Black/African American	9699 (21.4)	1430 (25.6)	7665 (21.4)		128 (18.6)	355 (13.4)		43 (20.2)	78 (18.0)			
Non-Hispanic White	23,077 (50.8)	1691 (30.3)	18,590 (51.9)		406 (58.8)	2008 (75.5)		104 (48.8)	278 (64.2)			
Native Hawaiian/Pacific Islander	194 (0.4)	49 (0.9)	100 (0.3)		8 (1.2)	18 (0.7)		8 (3.8)	11 (2.5)			
Hispanic	8791 (19.4)	1864 (33.4)	6598 (18.4)		100 (14.5)	155 (5.8)		38 (17.8)	36 (8.3)			
Other/unable to determine	1989 (4.4)	326 (5.8)	1572 (4.4)		26 (3.8)	45 (1.7)		10 (4.7)	10 (2.3)			
Payment source, n (%)				<.001			<.001			<.001	.257	<.001
Private	15,556 (34.4)	2284 (41.2)	11,820 (33.1)		297 (43.2)	913 (34.5)		92 (43.2)	150 (34.6)			
Veterans Affairs/CHAMPUS/Tricare	741 (1.6)	30 (0.5)	627 (1.8)		7 (1.0)	52 (2.0)		0 (0.0)	25 (5.8)			
Medicare	18,719 (41.4)	378 (6.8)	16,961 (47.5)		42 (6.1)	1161 (43.9)		11 (5.2)	166 (38.3)			
Medicaid	6490 (14.4)	1900 (34.3)	3970 (11.1)		211 (30.7)	276 (10.4)		78 (36.6)	55 (12.7)			
Not documented/other/self-pay	3720 (8.2)	954 (17.2)	2326 (6.5)		130 (18.9)	241 (9.1)		32 (15.0)	37 (8.6)			
Medical comorbidities												
Atrial fibrillation/flutter, n (%)	5034 (11.1)	51 (0.9)	4648 (13.0)	<.001	6 (0.9)	278 (10.5)	<.001	2 (0.9)	49 (11.3)	<.001	.992	<.001
Cancer, n (%)	5851 (12.9)	205 (3.7)	5193 (14.5)	<.001	21 (3.0)	329 (12.4)	<.001	10 (4.7)	93 (21.5)	<.001	.499	<.001
Cerebrovascular disease, n (%)	4909 (10.8)	148 (2.6)	4488 (12.5)	<.001	9 (1.3)	215 (8.1)	<.001	3 (1.4)	46 (10.6)	<.001	.059	<.001
Chronic kidney disease, n (%)	6281 (13.8)	235 (4.2)	5677 (15.8)	<.001	22 (3.2)	269 (10.1)	<.001	10 (4.7)	68 (15.7)	<.001	.406	<.001
Congenital heart disease, n (%)	122 (0.3)	28 (0.5)	84 (0.2)	<.001	3 (0.4)	6 (0.2)	.34	1 (0.5)	0 (0.0)	.15	.971	.6
Coronary artery disease, n (%)	5207 (11.5)	57 (1.0)	4741 (13.2)	<.001	7 (1.0)	353 (13.3)	<.001	1 (0.5)	48 (11.1)	<.001	.73	.42
Diabetes mellitus, n (%)	16,155 (35.6)	838 (15.0)	14,090 (39.3)	<.001	95 (13.8)	972 (36.6)	<.001	20 (9.4)	140 (32.3)	<.001	.058	<.001
Dyslipidemia, n (%)	16,776 (36.9)	300 (5.4)	15,353 (42.8)	<.001	32 (4.6)	941 (35.4)	<.001	8 (3.8)	142 (32.8)	<.001	.44	<.001
Heart failure, n (%)	5869 (12.9)	138 (2.5)	5276 (14.7)	<.001	17 (2.5)	364 (13.7)	<.001	9 (4.2)	65 (15.0)	<.001	.276	.34
Hypertension, n (%)	27,368 (60.3)	1031 (18.5)	24,340 (67.9)	<.001	133 (19.3)	1575 (59.2)	<.001	28 (13.1)	261 (60.3)	<.001	.117	<.001
Immune disorders, n (%)	2250 (5.0)	225 (4.0)	1843 (5.1)	<.001	27 (3.9)	112 (4.2)	.73	7 (3.3)	36 (8.3)	.02	.857	.001
Peripheral artery disease, n (%)	1377 (3.0)	11 (0.2)	1273 (3.6)	<.001	1 (0.1)	78 (2.9)	<.001	0 (0.0)	14 (3.2)	.01	.78	.24
Pulmonary embolism, n (%)	1282 (2.8)	94 (1.7)	1072 (3.0)	<.001	8 (1.2)	84 (3.2)	.004	8 (3.8)	16 (3.7)	.97	.037	.62
Pulmonary disease, n (%)	9736 (21.4)	812 (14.5)	8044 (22.4)	<.001	87 (12.6)	657 (24.7)	<.001	37 (17.4)	99 (22.9)	.11	.183	.03
Smoking, n (%)	3571 (7.9)	459 (8.2)	2587 (7.2)	.008	86 (12.5)	342 (12.9)	.78	25 (11.7)	72 (16.6)	.10	<.001	<.001

a CHAMP US, Civilian Health and Medical Program of the Uniformed Services; N/A, not applicable.

b Comparison *P* value compares young or older groups across all 3 time periods.

c Continuous variables presented as median (25^th^-75^th^ percentile). Continuous and categorical variables compared using Wilcoxon Rank-Sum test and χ^2^ tests, respectively.

When comparing admission characteristics, young and older patients were less likely to present with cough, fatigue, fevers or chills, headache, loss of taste or smell, nausea, vomiting, or diarrhea during omicron when compared with the other variant time periods. Patients in both age strata presenting during the delta period were more likely to present with shortness of breath, hypoxia, and with interstitial infiltrates on chest x-ray or computerized tomography (CT) when compared with the other periods. Older patients (more than 40 years) presented with increased rates of confusion or altered mental status, cough, fatigue, hypoxia, and interstitial infiltrates on chest x-ray or CT when compared with younger patients (18-40 years) across all variant time periods (Table 2).

**Table 2:** Hospital Admission Characteristics of the Cohort Stratified by Age and Coronavirus Disease 2019 Variant Time Period^a^

Characteristic	Overall	Wild type/alpha wave (January 20, 2020-July 5, 2021), N=41,426			Delta wave (July 6, 2021-December 27, 2021), n=3,349			Omicron wave (December 28, 2021-February 14, 2022), n=646			Comparison *P* value^b^	Comparison *P* value^b^
	(January 20, 2020-February 14, 2022)	Young (18-40) y	Older (>40) y	*P* value	Young (18-40) y	Older (>40) y	*P* value	Young (18-40) y	Older (>40) y	*P* value	Young (18-40) y	Older (>40) y
	N=45,421	n=5585	n=35841		n=690	n=2659		n=213	n=433			
Hospital presentation^c^												
Days from symptom onset to admission	5.0 (2.0-9.0)	5.0 (2.0-8.0)	5.0 (2.0-8.0)	.99	7.0 (3.0-9.0)	6.0 (3.0-10.0)	.99	1.0 (0.0-4.0)	3.0 (1.0-7.0)	<.001	<.001	<.001
Fever (temperature >38 °C), n (%)	7485 (16.9)	1035 (19.0)	5978 (17.1)	<.001	95 (13.9)	322 (12.2)	.23	17 (8.1)	38 (8.8)	.77	<.001	<.001
Tachycardia on admission (HR>100 beats/min), n (%)	14,435 (32.3)	2708 (49.3)	10,400 (29.5)	<.001	342 (49.7)	776 (29.2)	<.001	84 (39.4)	125 (28.9)	.007	.017	.93
Hypotension (systolic blood pressure<90 mm Hg), n (%)	1112 (2.5)	52 (1.0)	926 (2.7)	<.001	17 (2.5)	94 (3.5)	.16	6 (2.8)	17 (3.9)	.47	<.001	.009
Hypoxia (O_2_ Saturation <90% or requiring supplemental O_2_), n (%)	18,433 (42.4)	1407 (27.0)	14,769 (43.1)	<.001	362 (52.8)	1657 (62.4)	<.001	41 (19.5)	197 (45.5)	<.001	<.001	<.001
Interstitial infiltrates on chest x-ray or CT, n (%)	28,273 (66.5)	2756 (52.5)	23,081 (69.0)	<.001	392 (61.7)	1800 (71.5)	<.001	33 (15.5)	211 (48.7)	<.001	<.001	<.001
Admission symptoms												
Confusion or altered mental status, n (%)	4905 (11.4)	131 (2.5)	4427 (13.1)	<.001	15 (2.3)	289 (11.2)	<.001	3 (1.4)	40 (9.2)	<.001	.594	.002
Cough, n (%)	23,326 (54.2)	2669 (50.4)	18,808 (55.6)	<.001	309 (47.2)	1334 (51.7)	.04	56 (26.3)	150 (34.6)	.03	<.001	<.001
Fatigue, n (%)	12,675 (29.5)	1074 (20.3)	10,566 (31.2)	<.001	122 (18.6)	812 (31.4)	<.001	10 (4.7)	91 (21.1)	<.001	<.001	<.001
Fever/chills, n (%)	21,640 (50.3)	2706 (51.1)	17,503 (51.7)	.44	277 (42.3)	1012 (39.2)	.15	38 (17.8)	104 (24.0)	.08	<.001	<.001
Headache, n (%)	4465 (10.4)	704 (13.3)	3308 (9.8)	<.001	91 (13.9)	310 (12.0)	.19	13 (6.1)	39 (9.0)	.20	.008	.001
Loss of smell/taste, n (%)	2283 (5.3)	393 (7.4)	1692 (5.0)	<.001	44 (6.7)	147 (5.7)	.32	2 (0.9)	5 (1.2)	.80	.001	<.001
Myalgia, n (%)	8118 (18.9)	1136 (21.5)	6240 (18.4)	<.001	157 (24.0)	518 (20.1)	.03	19 (8.9)	48 (11.1)	.40	<.001	<.001
Nasal congestion, n (%)	2331 (5.4)	335 (6.3)	1733 (5.1)	<.001	41 (6.3)	173 (6.7)	.69	17 (8.0)	32 (7.4)	.79	.622	<.001
Nausea, vomiting, or diarrhea, n (%)	12,295 (28.6)	1573 (29.7)	9672 (28.6)	.09	200 (30.5)	715 (27.7)	.15	36 (16.9)	99 (22.9)	.08	<.001	.02
Shortness of breath, n (%)	25,920 (60.3)	2738 (51.7)	20,840 (61.6)	<.001	388 (59.2)	1740 (67.4)	<.001	42 (19.7)	171 (39.7)	<.001	<.001	<.001
Sore throat, n (%)	2191 (5.1)	410 (7.7)	1623 (4.8)	<.001	27 (4.1)	83 (3.2)	.25	15 (7.0)	33 (7.6)	.79	.004	<.001
Medication before admission												
Previous antiplatelet, n (%)	12,478 (28.7)	275 (5.1)	11,267 (33.0)	<.001	45 (6.5)	759 (28.6)	<.001	16 (7.5)	116 (26.8)	<.001	.106	<.001
Previous anticoagulant, n (%)	11,098 (30.1)	657 (14.9)	9262 (32.6)	<.001	108 (15.7)	936 (35.4)	<.001	21 (9.9)	114 (26.3)	<.001	.101	<.001
Previous antihypertensive, n (%)	23,973 (55.5)	847 (15.9)	21,215 (62.7)	<.001	112 (16.3)	1503 (56.6)	<.001	33 (15.5)	263 (60.7)	<.001	.952	<.001
Previous cholesterol lowering medication, n (%)	16,991 (39.4)	298 (5.6)	15,424 (45.6)	<.001	27 (3.9)	1049 (39.5)	<.001	10 (4.7)	183 (42.3)	<.001	.173	<.001
Previous antihyperglycemic, n (%)	11,988 (27.8)	598 (11.2)	10,437 (30.8)	<.001	77 (11.2)	741 (27.9)	<.001	16 (7.5)	119 (27.5)	<.001	.243	.002
Previous corticosteroid, n (%)	3954 (9.1)	341 (6.3)	3183 (9.3)	<.001	67 (9.7)	300 (11.3)	.24	15 (7.0)	48 (11.1)	.10	.004	.002
Previous immunosuppressive medicine (other than steroids), n (%)	1491 (3.4)	178 (3.3)	1150 (3.4)	.83	20 (2.9)	93 (3.5)	.44	9 (4.2)	41 (9.5)	.02	.633	<.001
Laboratory report												
White blood cell count (K/μL) on admission	7.1 (5.2-9.9)	7.5 (5.5-10.3)	7.0 (5.1-9.8)	<.001	7.4 (5.1-10.6)	7.5 (5.4-10.6)	.47	8.8 (6.2-11.7)	7.8 (5.2-11.0)	.001	<.001	<.001
Absolute lymphocyte count (×10^9^) on admission	1.0 (0.6-1.4)	1.2 (0.9-1.8)	0.9 (0.6-1.4)	<.001	1.0 (0.7-1.5)	0.9 (0.6-1.2)	<.001	1.3 (0.8-2.0)	1.0 (0.6-1.5)	<.001	<.001	<.001
Hemoglobin (g/dL) on admission	12.9 (11.3-14.3)	13.1 (11.5-14.6)	12.9 (11.3-14.2)	<.001	13.0 (11.5-14.6)	13.3 (11.7-14.7)	.24	12.2 (10.9-13.6)	12.5 (10.9-14.3)	.07	<.001	<.001
Platelets (K/μL) on admission	206.0 (158.0-270.0)	220 (176.0-279.0)	203 (156.0-267.0)	<.001	220.0 (164.0-286.0)	209.0 (158.0-281.5)	.10	244.5 (185.0-315.0)	212.0 (159.0-277.0)	<.001	.006	.003
Serum creatinine (mg/dL) on admission	1.0 (0.8-1.5)	0.8 (0.7-1.1)	1.1 (0.8-1.5)	<.001	0.8 (0.7-1.1)	1.0 (0.8-1.4)	<.001	0.7 (0.6-1.0)	1.1 (0.8-1.6)	<.001	.023	<.001
Peak troponin (ng/mL)	0.02 (0.01-0.05)	0.01 (0.003-0.03)	0.02 (0.01-0.05)	<.001	0.01 (0.01-0.02)	0.02 (0.01-0.07)	<.001	0.01 (0.01-0.03)	0.02 (0.01-0.06)	.02	.015	<.001
Peak b-type natriuretic peptide (ng/L)	72.0 (25.0-239.0)	23.0 (11.9-62.5)	78.0 (28.0-251.0)	<.001	27.0 (15.0-125.0)	81.0 (28.0-261.0)	.001	87.0 (13.0-802.0)	138.0 (41.0-461.0)	.67	.031	.007
Peak ferritin (ng/mL)	525.4 (234.0-1087.0)	393.0 (135.0-914.2)	541.0 (249.0-1107.9)	<.001	517.0 (220.5-1210.0)	581.0 (269.0-1273.7)	.48	35.0 (15.0-380.0)	341.0 (94.6-717.0)	.01	<.001	.001
Peak C-reactive protein (mg/L)	68.4 (22.0-133.3)	45.0 (11.7-105.0)	70.4 (23.0-134.0)	<.001	70.0 (33.0-139.0)	90.0 (47.0-178.0)	<.001	37.2 (10.0-91.0)	59.0 (23.5-138.0)	.17	<.001	<.001
Peak D-dimer (ng/mL)	920.0 (490.0-1840.0)	650.0 (364.0-1231.0)	970.0 (510.0-1930.0)	<.001	910.0 (512.0-2000.0)	870.0 (452.0-1850.0)	.40	927.5 (601.0-2039.0)	714.0 (386.0-1648.0)	.13	<.001	.004
Peak procalcitonin (ng/mL)	0.1 (0.1-0.4)	0.1 (0.1-0.3)	0.1 (0.1-0.4)	<.001	0.1 (0.1-0.5)	0.2 (0.1-0.5)	.09	0.1 (0.1-0.2)	0.2 (0.1-0.6)	.11	.011	<.001

a CT, computed tomography; HR, heart rate; Med, medication; O_2_, oxygen.

b Comparison *P* value compares young or older groups across all 3 time periods.

c Continuous variables presented as median (25th-75th percentile). Continuous and categorical variables compared using Wilcoxon Rank-Sum test and χ^2^ tests, respectively.

When evaluating therapies received during hospitalization, patients in both age strata presenting during the wild type/alpha period were more likely to be treated with convalescent serum, hydroxychloroquine, and azithromycin, whereas patients presenting during the delta period were more likely to be treated with mechanical ventilation, inotropes or vasopressors, corticosteroids, remdesivir, and tocilizumab when compared with the other variant time periods (Table 3). When comparing age groups, older patients were more likely to receive corticosteroids, remdesivir, tocilizumab, mechanical ventilation, and inotropes or vasopressors when compared with younger patients across all 3 periods. In univariate analysis, older patients aged more than 40 years presenting during delta were found to experience more acute myocardial infarction, deep vein thrombosis or pulmonary embolism, in-hospital shock, and in-hospital mortality when compared with the other time periods. Younger patients (18-40 years) were more likely to experience deep vein thrombosis or pulmonary embolism and in-hospital mortality during the delta period compared with the other waves. Rates of myocarditis and new-onset heart failure were low across all 3 variants. (Table 3). Rates of missingness are shown in the Supplemental Table, available online at http://www.mcpiqojournal.org.

**Table 3:** Hospitalization Characteristics and Outcomes of the Cohort by Age and Coronavirus Disease 2019 Variant Time Period^a^

Characteristic/outcomes	Overall	Wild type/alpha wave (January 20, 2020-July 5, 2021), n=41,426			Delta wave (July 6, 2021-December 27, 2021), n=3,349			Omicron wave (December 28, 2021-February 14, 2022), n=646			Comparison *P* value^b^	Comparison *P* value^b^
	(January 20, 2020-February 14, 2022)	Young (18-40) y	Older (>40) y	*P* value	Young (18-40) y	Older (>40) y	*P* value	Young (18-40) y	Older (>40) y	*P* value	Young (18-40) y	Older (>40) y
	N=45,421	n=5585	n=35841		n=690	n=2659		n=213	n=433			
Therapies received during hospitalization^c^												
Corticosteroids, n (%)	24,018 (53.9)	2006 (36.9)	19,096 (54.3)	<.001	464 (67.3)	2147 (81.0)	<.001	59 (27.7)	246 (56.8)	<.001	<.001	<.001
Immunoglobulins, n (%)	277 (0.7)	31 (0.6)	220 (0.7)	.85	1 (0.2)	25 (1.0)	.03	0 (0.0)	0 (0.0)	N/A	.014	.003
Convalescent serum, n (%)	4438 (10.4)	346 (6.6)	4037 (12.0)	<.001	8 (1.3)	47 (1.9)	.13	0 (0.0)	0 (0.0)	N/A	<.001	<.001
Ritonavir/lopinavir, n (%)	204 (0.5)	22 (0.4)	182 (0.5)	.07	0 (0.0)	0 (0.0)	N/A	0 (0.0)	0 (0.0)	N/A	.055	.001
Hydroxychloroquine, n (%)	8144 (18.7)	852 (15.9)	7269 (21.3)	<.001	4 (0.6)	14 (0.5)	.18	2 (0.9)	3 (0.7)	.74	<.001	<.001
Azithromycin, n (%)	14,218 (32.6)	1531 (28.5)	11,744 (34.3)	<.001	153 (22.2)	722 (27.2)	.02	9 (4.2)	59 (13.6)	.001	<.001	<.001
Remdesivir, n (%)	14,631 (32.8)	1199 (22.1)	11,712 (33.3)	<.001	284 (41.2)	1328 (50.1)	<.001	14 (6.6)	94 (21.7)	<.001	<.001	<.001
Tocilizumab, n (%)	3053 (7.0)	251 (4.7)	2486 (7.3)	<.001	60 (8.7)	242 (9.1)	.008	1 (0.5)	13 (3.0)	.04	<.001	<.001
Anticoagulation, n (%)	11,098 (30.1)	657 (14.9)	9262 (32.6)	<.001	108 (15.7)	936 (35.4)	<.001	21 (9.9)	114 (26.3)	<.001	.101	<.001
Procedures performed during hospitalization												
Mechanical ventilation, n (%)	7515 (17.2)	590 (11.0)	6237 (18.2)	<.001	105 (15.2)	518 (19.5)	.01	14 (6.6)	51 (11.8)	.04	<.001	<.001
Use of inotropes/vasopressors, n (%)	3754 (8.3)	223 (4.0)	3193 (8.9)	<.001	35 (5.1)	272 (10.2)	<.001	5 (2.4)	26 (6.0)	.05	.202	.02
Use of mechanical circulatory support, n (%)	196 (0.5)	35 (0.6)	155 (0.4)	.05	3 (0.4)	3 (0.1)	.11	0 (0.0)	0 (0.0)	N/A	.74	.01
Outcomes during hospitalization												
Acute myocardial infarction, n (%)	1521 (3.5)	44 (0.8)	1301 (3.8)	<.001	8 (1.2)	146 (5.5)	<.001	1 (0.5)	21 (4.9)	.004	.54	<.001
Cardiac Arrest, n (%)	1678 (3.9)	84 (1.6)	1470 (4.3)	<.001	16 (2.3)	101 (3.8)	.06	0 (0.0)	7 (1.6)	.10	.054	.01
Deep vein thrombosis or pulmonary embolus, n (%)	1926 (4.2)	135 (2.4)	1533 (4.3)	<.001	28 (4.1)	202 (7.6)	.001	4 (1.9)	24 (5.5)	.04	.03	<.001
In-hospital mortality, n (%)	6339 (14.0)	161 (2.9)	5644 (15.7)	<.001	40 (5.8)	455 (17.1)	<.001	1 (0.5)	38 (8.8)	<.001	<.001	<.001
In-hospital shock, n (%)	4604 (10.7)	280 (5.2)	3875 (11.4)	<.001	48 (7.0)	347 (13.1)	<.001	11 (5.2)	43 (10.0)	.05	.166	.02
Ischemic stroke/intracranial hemorrhage, n (%)	783 (1.8)	34 (0.6)	631 (1.8)	<.001	6 (0.9)	83 (3.1)	.001	3 (1.4)	26 (6.0)	.008	.33	<.001
Myocarditis, n (%)	102 (0.2)	20 (0.4)	78 (0.2)	.05	2 (0.3)	2 (0.1)	.15	0 (0.0)	0 (0.0)	N/A	.641	.16
New-onset heart failure, n (%)	852 (2.0)	41 (0.8)	747 (2.2)	<.001	4 (0.6)	50 (1.9)	.02	0 (0.0)	10 (2.3)	.04	.392	.57
New hemodialysis/renal replacement therapy, n (%)	1578 (3.6)	106 (2.0)	1352 (4.0)	<.001	13 (1.9)	95 (3.6)	.03	2 (0.9)	10 (2.3)	.35	.561	.14
Seizure, n (%)	337 (0.8)	41 (0.8)	266 (0.8)	.89	5 (0.7)	17 (0.6)	.80	2 (0.9)	6 (1.4)	1	.951	.25
Discharge disposition												
Home, n (%)	29,229 (64.4)	5015 (89.8)	21,312 (59.5)	<.001	586 (84.9)	1791 (67.4)	<.001	203 (95.3)	322 (74.4)	<.001	<.001	<.001
Hospice (home or health care facility), n (%)	1270 (2.8)	11 (0.2)	1188 (3.3)	<.001	1 (0.1)	54 (2.0)	<.001	1 (0.5)	15 (3.5)	.02	.644	.001
Acute care facility or other health care facility, N (%)	8145 (17.9)	313 (5.6)	7435 (20.7)	<.001	36 (5.2)	307 (11.5)	<.001	2 (0.9)	52 (12.0)	<.001	.012	<.001
Expired, n (%)	6314 (13.9)	161 (2.9)	5622 (15.7)	<.001	40 (5.8)	452 (17.0)	<.001	1 (0.5)	38 (8.8)	<.001	<.001	<.001
Other, n (%)	463 (1.0)	85 (1.5)	284 (0.8)	<.001	27 (3.9)	55 (2.1)	.005	6 (2.8)	6 (1.4)	.21	<.001	<.001

a N/A, not applicable.

b Comparison *P* value compares young or older groups across all 3 time periods.

c Continuous variables presented as median (25th-75th percentile). Continuous and categorical variables compared using Wilcoxon Rank-Sum test and χ^2^ tests, respectively.

In adjusted logistic regression models evaluating the association of COVID-19 variant time period on in-hospital outcomes among patients 18-40 years, patients presenting during the delta period had increased odds of severe COVID-19 (odds ratio [OR], 1.64; 95% CI, 1.29-2.08), MACE (OR, 1.76; 95% CI, 1.25-2.49), and in-hospital mortality (OR, 2.24; 95% CI, 1.51-3.32), and a decreased odds of discharge to home (OR, 0.69; 95% CI, 0.54-0.89) when compared with patients presenting during the wild type/alpha period. Young patients aged 18 to 40 years presenting during the omicron period were not found to have different odds of severe COVID, MACE, in-hospital mortality, or thromboembolic disease when compared with those presenting during the wild type/alpha period (Table 4). Among older patients (aged more than 40 years), patients presenting during the delta period were found to have increased odds of severe COVID-19 (OR, 1.19; 95% CI, 1.08-1.31), MACE (OR, 1.54; 95% CI, 1.39-1.71), thromboembolic disease (OR, 1.79; 95% CI, 1.53-2.11), in-hospital mortality (OR, 1.44; 95% CI, 1.29-1.62), and discharge to home (OR, 1.15; 95% CI, 1.05-1.27) when compared with patients presenting during the wild type/alpha period. Older patients aged more than 40 years presenting during the omicron period had decreased odds of severe COVID-19 (OR, 0.66; 95% CI, 0.51-0.87) and in-hospital mortality (OR, 0.64; 95% CI, 0.45-0.91) but not MACE or thromboembolic disease when compared with patients presenting during the wild type/alpha period (Table 4).

**Table 4:** Association of COVID-19 Variant Time Period with Outcomes Among Patients 18-40 Years and >40 Years Presenting with COVID-19^a^^,^^b^

Age group: 18-40 y									
Outcome	Wild type/alpha		Delta			Omicron			*P* value^c^
	N event/no event	Ref.	N event/no event	Odds ratio (95% CI)	*P* value	N event/no event	Odds ratio (95% CI)	*P* value	
Severe COVID-19	606/4979	1	108/582	1.64 (1.29-2.08)	<.0001	14/199	0.66 (0.37-1.18)	.16	<.0001
Major adverse cardiovascular events	252/5,333	1	51/639	1.76 (1.25-2.49)	.001	5/208	0.66 (0.26-1.63)	.37	.003
In-hospital mortality	161/5424	1	40/650	2.24 (1.51-3.32)	<.0001	1/212	0.21 (0.03-1.55)	.13	<.0001
Thromboembolic disease (DVT or PE)	135/5450	1	28/662	1.77 (1.14-2.75)	.01	4/209	0.63 (0.19-2.04)	.44	.03
Discharge to home	5015/570	1	586/104	0.69 (0.54-0.89)	.004	203/10	2.32 (1.17-4.59)	.02	.0005

Age group: >40 y									
Outcome	Wild type/alpha		Delta			Omicron			*P* value^c^
	N event/no event	Ref.	N event/no event	Odds ratio (95% CI)	*P* value	N event/no event	Odds ratio (95% CI)	*P* value	
Severe COVID-19	8527/27,314	1	641/2018	1.19 (1.08-1.31)	.0006	66/367	0.66 (0.51-0.87)	.003	<.0001
Major adverse cardiovascular events	7231/28,610	1	625/2034	1.54 (1.39-1.71)	<.0001	81/352	1.12 (0.87-1.44)	.38	<.0001
In-hospital mortality	5644/30,197	1	455/2204	1.44 (1.29-1.62)	<.0001	38/395	0.64 (0.45-0.91)	.01	<.0001
Thromboembolic disease (DVT or PE)	1533/34,308	1	202/2457	1.79 (1.53-2.11)	<.0001	24/409	1.19 (0.77-1.84)	.43	<.0001
Discharge to home	21,312/14,529	1	1791/868	1.15 (1.05-1.27)	.003	322/111	1.80 (1.42-2.27)	<.0001	<.0001

a CI, confidence interval; COVID-19, coronavirus disease 2019; DVT, deep vein thrombosis; PE, pulmonary embolism; Ref., reference.

b Models adjusted for age, sex, body mass index, race, payment source, and medical comorbidities (atrial fibrillation or flutter, cancer, cerebrovascular disease, chronic kidney disease, congenital heart disease, coronary artery disease, diabetes mellitus, dyslipidemia, heart failure, hypertension, immune disorders, peripheral artery disease, pulmonary embolism, pulmonary disease, and smoking).

c *P* value for overall COVID-19 wave effect; Wald χ^2^ test.

Table 5 evaluates the association of age with outcomes across the different COVID-19 variant time periods. During the overall pandemic, compared with young patients (aged 18-40 years), older patients (aged more than 40 years) were found to have increased odds of severe COVID-19 (OR, 1.88; 95% CI, 1.71-2.06), MACE (OR, 3.08; 95% CI, 2.70-3.51), thromboembolic disease (OR, 2.29; 95% CI, 1.91-2.74), and in-hospital mortality (OR, 3.60; 95% CI, 3.06-4.23), and decreased odds of discharge to home (OR, 0.37; 95% CI, 0.34-0.41). Compared with young patients, older patients were found to have increased odds of severe COVID-19, MACE, thromboembolic disease, and in-hospital mortality across both wild type/alpha and delta variant time periods. During omicron, compared with young patients, older patients were further found to have increased odds of MACE, thromboembolic disease, and in-hospital mortality (Table 5).

**Table 5:** Association of Age Group (Age>40 vs 18-40) with Outcomes Among Patients Presenting with COVID-19 by Variant Time Period^a^^,^^b^

Outcome^c^	Overall		Wild type/alpha		Delta		Omicron	
	OR (95% CI)	*P* value	OR (95% CI)	*P* value	OR (95% CI)	*P* value	OR (95% CI)	*P* value
Severe COVID-19	1.88 (1.71-2.06)	<.0001	1.93 (1.74-2.13)	<.0001	1.67 (1.29-2.16)	.0001	1.74 (0.81-3.74)	.16
Major adverse cardiovascular events	3.08 (2.70-3.51)	<.0001	3.13 (2.71-3.62)	<.0001	2.96 (2.12-4.15)	<.0001	4.63 (1.66-12.93)	.004
In-hospital mortality	3.60 (3.06-4.23)	<.0001	3.86 (3.21-4.61)	<.0001	2.69 (1.85-3.93)	<.0001	10.07 (1.22-83.39)	.03
Thromboembolic disease (DVT or PE)	2.29 (1.91-2.74)	<.0001	2.23 (1.83-2.72)	<.0001	2.65 (1.70-4.13)	<.0001	5.83 (1.37-24.75)	.02
Discharge to home	0.37 (0.34-0.41)	<.0001	0.36 (0.32-0.40)	<.0001	0.50 (0.38-0.65)	<.0001	0.26 (0.12-0.59)	.001

a CI, confidence interval; COVID-19, coronavirus disease 2019; DVT, deep vein thrombosis; PE, pulmonary embolism.

b Models adjusted for sex, body mass index, race, payment source, and medical comorbidities (atrial fibrillation or flutter, cancer, cerebrovascular disease, chronic kidney disease, congenital heart disease, coronary artery disease, diabetes mellitus, dyslipidemia, heart failure, hypertension, immune disorders, peripheral artery disease, pulmonary embolism, pulmonary disease, and smoking)

c Regression models compare outcomes of adults (>40 years) to young adults (≤40 years)

**Figure 1:**
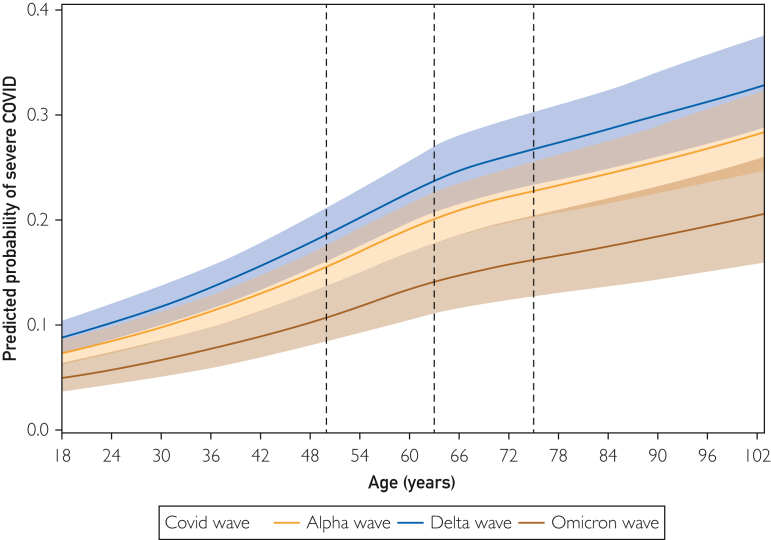
Splines reporting association of continuous age with predicted probability of severe coronavirus disease 2019 (COVID-19).

**Figure 2:**
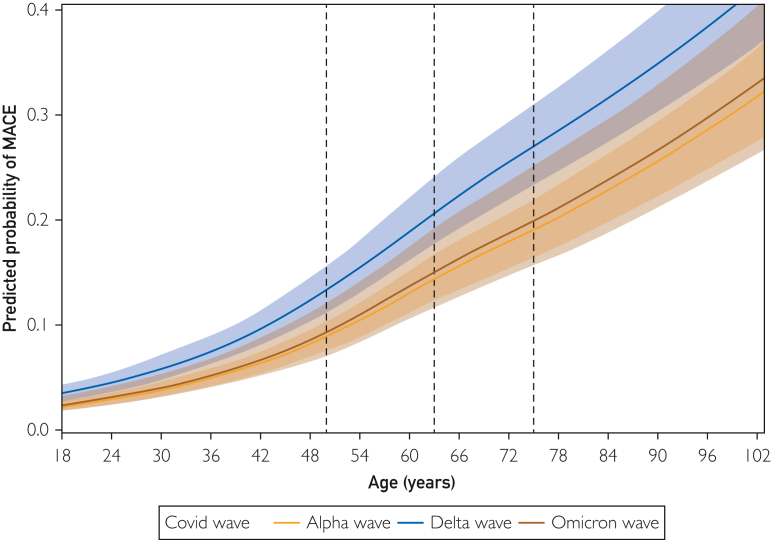
Splines reporting association of continuous age with predicted probability of major adverse cardiovascular events (MACE).

**Figure 3:**
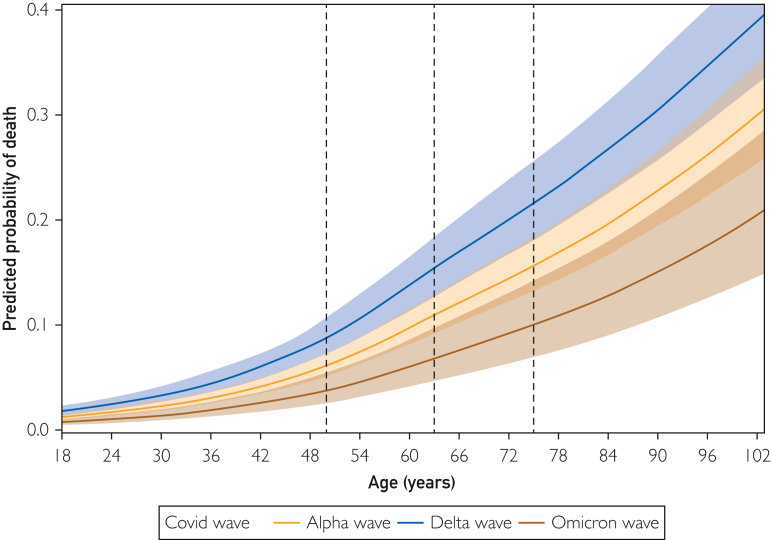
Splines reporting association of continuous age with predicted probability of death.

In adjusted spline models continuously modeling the impact of age, increasing age was found to associate with increased predicted probability of severe COVID-19, MACE, and in-hospital mortality across the wild type/alpha, delta, and omicron time periods (Figure 1, Figure 2, Figure 3).

## Discussion

Using the national AHA GWTG COVID-19 cardiovascular disease registry database of 45,421 patients admitted with COVID-19 between January 20, 2020 and February 14, 2022, we compared patient characteristics and outcomes stratified by age across the 3 main COVID-19 variant time periods in the United States. In both young (aged 18 to 40 years) and older adults (aged more than 40 years), those hospitalized with COVID-19 during a period of delta predominance were found to have increased odds of severe COVID-19, MACE, thromboembolic disease, and in-hospital mortality when compared with those presenting during wild type/alpha. Among older patients (aged more than 40 years), those presenting during a time of omicron predominance were found to have decreased odds of severe COVID-19 and in-hospital mortality when compared with wild type/alpha. Across all 3 variant time periods, increasing age was associated with an increased predicted probability of severe COVID-19, MACE, and in-hospital mortality.

### COVID-19 Evolution

The initial strain of COVID-19 (wildtype) was first discovered in Wubei Province, China in December 2019.^1^ The virus itself was found to have 90 homotrimeric spike receptors on its membrane, with a mechanism of infectivity involving spike protein binding to the angiotensin converting enzyme 2 (ACE2) receptor on host cells.^12^ As the wild type strain spread around the world, it began to acquire mutations, changing both its infectivity and severity patterns. Strains with mutations became known as variants, with alpha, delta, and omicron being the 3 main variants to date in the United States.^3^ The alpha variant was found to have over 12 main mutations in its spike protein, including 7 amino acid substitutions and 2 deletions.^12^^,^^13^ These mutations were shown to increase binding affinity, cell entry, infectivity, and transmissibility.^12^ The delta variant was found to have further spike protein mutations with resultant increased transmissibility, viral load, and ultimate ability to evade CD8 T cells.^6^ Last, the omicron variant was found to possess a marked degree of sequence variation, with at least 32 mutations in the spike protein alone.^5^^,^^12^

These genetic mutations have created distinct patterns of transmissibility, infectivity, and severity, and indeed, previous studies have shown clinical differences between the different COVID-19 strains. The delta variant, compared with the initial wild type or alpha strains, has been shown to spread more easily, cause more severe disease, have a greater risk of hospitalization and intensive care unit admission, and has an increased risk of death.^6^^,^^14^, ^15^, ^16^, ^17^ When comparing omicron to delta, studies have found increased infectivity through lower clinical severity, a lower duration of symptoms, and a lower risk of hospital admission or health care service utilization, intensive care unit admission, mechanical ventilation, and death.^4^^,^^5^^,^^7^^,^^18^, ^19^, ^20^, ^21^, ^22^, ^23^, ^24^

### Clinical Presentation, Treatment Patterns, and Outcomes

These previous findings are consistent with the results of our study. For example, patients of both age strata presenting during the delta period were more likely to present with hypoxia, dyspnea, and interstitial infiltrates when compared with the other strains, whereas those presenting during omicron were found to present with milder symptoms such as nasal congestion and were less likely to present with loss of smell or taste. With regards to treatments received during hospitalization, those admitted during the delta period were more likely to receive mechanical ventilation, corticosteroids, remdesivir, and tocilizumab when compared with the other variants. In adjusted models, both younger and older patients presenting during delta had increased odds of severe COVID-19, MACE, thromboembolic disease, and in-hospital mortality when compared with wild type/alpha. Among patients aged more than 40 years, those presenting during omicron were shown to have decreased odds of severe COVID-19 and in-hospital mortality when compared to those presenting during wild type/alpha.

The differences observed in clinical presentation, treatments received, and outcomes may in part be due to the virulence and location of predominant viral replication in the different variants. In laboratory studies, for example, omicron has been shown to replicate more in the upper airways and less in the lungs and may cause a milder form of disease.^25^^,^^26^ Decreased severity in symptomology and outcomes during omicron may also be attributed to greater rates of vaccination in those presenting during the omicron time period.^21^ One study from California reported that a greater proportion of patients admitted with COVID-19 during omicron were fully vaccinated (according to Centers for Disease Control definitions at the time) when compared to a period of delta predominance (39.6% vs 25.1%).^21^ There were also fewer unvaccinated patients hospitalized during omicron when compared with delta (56.4% vs 71.1%).^21^ Finally, previous studies have reported increasing percentages of hospitalized patients during omicron admitted for an alternate diagnosis who were found to incidentally have COVID-19, which may further explain the improved clinical severity and outcomes in this group.^27^^,^^28^

When stratifying the cohort by age, we found increased odds of adverse outcomes in those presenting during the delta period compared with wild type/alpha among both young patients (age 18-40 years) and older patients (age more than 40 years). When evaluating the impact of age across each variant time period, we found increased odds of adverse outcomes among older patients compared with younger patients across all 3 variant time periods. Last, when continuously modeling age as a predictor of adverse outcomes, we show that increasing age associated with an increased predicted probability of severe COVID-19, MACE, and in-hospital mortality. Previous studies support these findings and generally report a relationship between increasing age and increased risk of COVID-19 morbidity and mortality.^8^^,^^29^, ^30^, ^31^, ^32^, ^33^, ^34^, ^35^ Several reasons have been postulated for this association and include increased basal inflammation, hyperresponsiveness of immune cells, ineffective T cell priming, decreased T cell diversity, diminished antibody response or activity, and an unregulated innate immune system in those of older age.^36^ A higher prevalence of comorbidities, differential host receptor expression, and variations in coagulopathy have also been suggested to play a role.^29^^,^^32^^,^^37^

### Limitations

Data included in this study are from voluntary participating institutions in the GWTG COVID-19 cardiovascular disease registry, and therefore may not be fully generalizable to the overall United States population. Fewer patients were enrolled during the delta and omicron periods from a smaller number of participating sites, which may further limit generalizability. We are only able to determine the time period during which patients were hospitalized with COVID-19, and so we are unable to determine the strain of the virus infecting the patient. The data gathered are observational, and therefore, causality cannot be established. The data and outcomes are only gathered from the patient’s in-patient admission. Post discharge outcomes are not available. The vaccination rates of the cohort were not tracked. Although logistic regression and spline models were adjusted for possible confounders, residual confounding may still exist.

## Conclusion

In one of the largest national COVID-19 analyses to date, we describe demographic, comorbidities, clinical characteristics, hospital treatment patterns, and outcomes for 45,421 patients admitted with COVID-19 in the United States between January 20, 2020 and February 14, 2022, stratified by patient age. Patients presenting during a period of delta predominance were found to have increased morbidity and mortality, whereas patients aged more than 40 years presenting during omicron reported decreased outcome severity when compared with those presenting during wild type/alpha. Increasing age adversely associated with outcomes across all 3 COVID-19 variant time periods. These data provide an important snapshot into the clinical characteristics and outcomes of hospitalized COVID-19 patients stratified by age during the first 2 years of the pandemic in the United States.

## Potential Competing Interests

Dr Fonarow reports consulting for Abbott, Amgen, AstraZeneca, Bayer, Cytokinetics, Edwards, Eli Lilly, Janssen, Medtronic, Merck, and Novartis. Dr Parikh receives research support from the American Heart Association, Janssen, Infraredx, Abbott Vascular, and Bayer, and consulting fees from Abbott Vascular. Dr de Lemos reports consulting income from Eli Lilly, Novo Nordisc, and Astra Zeneca. Dr Yang reports research grants/funding from CSL Behring, Boehringer Ingelheim, Eli Lilly, and Bristol Meyers Squibb, and consulting fees from Pfizer.
