# Supplementary material for: Impact of Age and Variant Time Period on Clinical Presentation and Outcomes of Hospitalized Coronavirus Disease 2019 Patients
Source: Mayo Clin Proc Innov Qual Outcomes. 2023 Sep 15;7(5):411–29. doi: 10.1016/j.mayocpiqo.2023.07.004 (PMC10507578; doi:10.1016/j.mayocpiqo.2023.07.004)
Supplement: Supplemental Data [file mmc1.pdf]

Supplementary Table 1. Missingness of the Cohort

	Wild Type/Alpha Missing N	Delta Missing N	Omicron Missing N
**Demographics**			
Age	0	0	0
Female	0	0	0
Body Mass Index	3382	165	22
Race	1	0	0
Asian			
American Indian/Alaska Native			
Non-Hispanic Black or African American			
Non-Hispanic White			
Native Hawaiian or Pacific Islander			
Hispanic			
Other/Unable to Determine			
Payment Source	176	19	0
Private			
Veterans Affairs/CHAMPUS/Tricare			
Medicare			
Medicaid			
Not Documented/Other/Self Pay			
**Medical Comorbidities**			
Atrial Fibrillation/Flutter	0	0	0
Cancer	0	0	0
Cerebrovascular Disease	0	0	0
Chronic Kidney Disease	0	0	0
Congenital Heart Disease	0	0	0
Coronary Artery Disease	0	0	0
Diabetes Mellitus	0	0	0
Dyslipidemia	0	0	0
Heart Failure	0	0	0
Hypertension	0	0	0
Immune Disorders	0	0	0
Peripheral Artery Disease	0	0	0
Pulmonary Embolism	0	0	0
Pulmonary Disease	0	0	0
Smoking	0	0	0
**Hospital Presentation**			
Days from Symptom Onset to Admission	10886	1052	179
Fever (Temperature >38C)	1002	16	7

Tachycardia on Admission (HR>100 beats per minute)	685	8	1
Hypotension (Systolic Blood Pressure < 90 mm Hg)	1352	10	1
Hypoxia (Oxygen saturation <90% or requiring supplemental O2)	1945	8	3
Interstitial Infiltrates on Chext X-Ray or CT	2740	197	0
**Admission Symptoms**			
Confusion or Altered Mental Status	2293	112	0
Cough	2293	112	0
Fatigue	2293	112	0
Fever/Chills	2293	112	0
Headache	2293	112	0
Loss of Smell/Taste	2293	112	0
Myalgia	2293	112	0
Nasal Congenstion	2293	112	0
Nausea, Vomiting, or Diarrhea	2293	112	0
Shortness of Breath	2293	112	0
Sore Throat	2293	112	0
**Medication Prior to Admission**			
Prior Antiplatelet	1877	2	0
Prior Anticoagulant	8555	14	0
Prior Antihypertensive	2244	3	0
Prior Cholesterol Lowering Medication	2244	3	0
Prior Anti-hyperglycemic	2253	2	0
Prior Corticosteroid	1873	2	0
Prior Immunosuppressive Medication (other than steroids)	1875	2	0
**Labs**			
White Blood Cell Count on admission	1917	711	17
Absolute Lymphocyte Count on admission	5753	1139	127
Hemoglobin on admission	1833	701	9
Platelets on admission	1823	711	11
Serum Creatinine on admission	2514	838	67
Peak Troponin	14413	1911	374
Peak B-Type Natriuretic Peptide	34289	2829	528
Peak Ferritin	19342	2645	576
Peak C-Reactive Protein	17712	2167	497
Peak Interleukin-6	37012	3336	646
Peak D-Dimer	22515	2509	533
Peak Procalcitonin	19716	2594	582
**Therapies Received During Hospitalization**			
Corticosteroids	830	9	0

Immunoglobulins	2763	204	0
Convalescent Serum	2613	204	0
Ritonavir/Lopinavir	2344	9	0
Hydroxychloroquine	1950	9	0
Azithromycin	1802	9	0
Remdesivir	863	9	0
Tocilizumab	1921	10	0
Anticoagulation	8555	14	0
**Procedures Performed During Hospitalization**			
Mechanical Ventilation	1757	5	0
Use of Inotropes/Vasopressors	1752	4	0
Use of Mechanical Circulatory Support	1752	4	0
**Outcomes During Hospitalization**			
Acute Myocardial Infarction	1901	5	0
Cardiac Arrest	1905	4	1
Death	0	0	0
Deep Vein Thrombosis or Pulmonary Embolus	0	0	0
In-Hospital Shock	2187	3	1
Ischemic Stroke/Intracranial Hemorrhage	1901	4	1
Myocarditis	1904	3	1
New-Onset Heart Failure	1891	3	1
New Hemodialysis or Renal Replacement Therapy	1857	5	1
Seizure	1908	4	1
**Discharge Disposition**			
Home	0	0	0
Hospice (Home or Healthcare Facility)	0	0	0
Acute Care Facility or Other Healthcare Facility	0	0	0
Expired	0	0	0
Other	0	0	0
